# Valorization of Saithe (*Pollachius virens*) Residuals into Protein Hydrolysates—Silaging as Preservation Technology

**DOI:** 10.3390/foods13132133

**Published:** 2024-07-04

**Authors:** Line Skontorp Meidell, Rasa Slizyte, Revilija Mozuraityte, Ana Karina Carvajal, Turid Rustad, Eva Falch

**Affiliations:** 1 Department of Biotechnology and Food Science, Norwegian University of Science and Technology (NTNU), 7012 Trondheim, Norway; turid.rustad@ntnu.no (T.R.); eva.falch@ntnu.no (E.F.); 2 Department of Fisheries and New Biomarine Industry, SINTEF Ocean, 7010 Trondheim, Norway; rasa.slizyte@sintef.no (R.S.); revilija.mozuraityte@sintef.no (R.M.); ana.k.carvajal@sintef.no (A.K.C.)

**Keywords:** saithe, residual raw material, valorization, quality, freshness, sorting, silage, protein hydrolysate

## Abstract

Silaging can be used as preservation technology to valorize currently discarded raw material into protein hydrolysate on board deep-sea vessels. The aim of this study was to investigate the effect of sorting and raw material freshness on the quality and yield of protein hydrolysates obtained through silaging of saithe (*Pollachius virens*) viscera. Additionally, the effect of using acid-containing antioxidants was tested. Out sorting of the liver prior to silaging resulted in slightly higher hydrolysate yields. The hydrolysates with the highest protein contents were obtained from silages made from fresh raw materials (day 0), and the content decreased significantly after longer storage of the raw material (2–3 days at 4 °C). Storage of the raw material for 1 day did not affect the quality. However, a significantly higher degree of hydrolysis (DH), content of free amino acids (FAA), and total volatile basic nitrogen (TVB-N) were obtained when raw materials were stored for 3 days. The FAA composition was influenced by the raw material’s freshness, with increases in free glutamic acid and lysine and a decrease in free glutamine after longer storage. None of the studied parameters were significantly affected by out sorting of liver or the addition of antioxidants. Overall, the results indicate that the whole fraction of the viscera can be utilized without reducing the quality of the hydrolysate and that the raw material should be stored for a maximum of 1 day prior to preservation to optimize the quality.

## 1. Introduction

Valorization of residual raw material is necessary to promote sustainable food systems. As one of the world’s most important seafood nations [1], Norway has a special responsibility to improve sustainability in seafood value chains. However, more than 147,000 tons of residual raw material was not utilized in the Norwegian seafood industry in 2022 [2]. This includes raw material that was not the primary product after gutting and processing [3]. The most significant part of unutilized raw material was generated on board deep-sea vessels during fishing of saithe (*Pollachius virens*), Atlantic cod (*Gadus morhua*), and haddock (*Melanogrammus aeglefinus*) [2]. In this segment of the fishing fleet, almost 70% of the residual raw material was discarded at sea. Viscera constituted the most important fraction. This raw material consists of internal organs such as stomach, intestines, liver, and gonad, which contain valuable nutrients, including proteins with excellent amino acid profiles, peptides, health beneficial omega-3 fatty acids, minerals, and vitamins that can be upgraded to food and feed ingredients [4,5]. However, the viscera is especially prone to fast quality degradation due to high enzymatic and microbial activity [3,6]. Additionally, high contents of polyunsaturated fatty acids (PUFAs), water, and organic compounds make the raw material susceptible to lipid oxidation and microbial spoilage [7,8,9]. Due to the highly unstable nature of the raw material, optimal onboard strategies for handling, preservation, and storage are necessary to enable its valorization [10].

Silaging is a preservation technology that can be implemented on board deep-sea vessels [2]. It has long traditions within seafood processing, where the low pH (that is kept below 4) controls the microbiota and the resulting silage can be stored for several weeks [11]. During the process, endogenous enzymes in the raw material hydrolyze protein into peptides and free amino acids, and after a certain period of storage, the soluble protein hydrolysate (stick water) can be separated and evaporated into protein hydrolysate [12]. The hydrolysate is regarded as a nutrient-rich product for animal feeding [13]. In addition, a recent study by Sajib et al. [14] suggested that silage hydrolysate made from viscera containing raw material from herring could serve as a good protein source in food, containing all essential amino acids. However, silage and silage-derived hydrolysates are often seen as low-quality products due to the high content of free amino acids [15]. As the application of silaging as a preservation technology is limited by the quality of the produced ingredients, there is a need to investigate possibilities for quality optimization.

Since most of the lipids in whitefish are located in the liver, sorting out the liver for thermal production of liver oil can be an option [9]. The lipid content in the raw material may influence the quality of the protein hydrolysate, as lipids and lipid oxidation products tend to form complexes with free amino acids and proteins, promote protein oxidation, and influence proteolytic activity and functional properties [16]. At the same time, preservation of the whole viscera fraction might be necessary, as sorting operations are resource-demanding [17], and there are situations in which the liver quality is not sufficiently good for oil production. However, to the best of our knowledge, no published studies have investigated the effect of out sorting of liver on the quality of protein hydrolysate obtained from silaging whitefish viscera.

Onboard silaging faces different challenges compared to onshore processing. Significant variation in catch size and onboard processing capacity might influence the time from catch to processing, resulting in degradation of the highly perishable viscera [18]. While several studies have investigated the effect of silaging time on the quality of the silage product [19,20,21,22,23,24,25], little is known about the effect of raw material freshness on the remaining protein hydrolysate.

To gain a better understanding of these aspects, this study aimed to evaluate the effects of out sorting of liver and raw material freshness on the yield and quality of protein hydrolysate obtained by silaging of saithe viscera. In addition, silaging with a commonly used antioxidant mixture (propyl gallate, citric acid, propylene glycol and butylated hydroxyanisole; BHA) was tested. Measurements of proteolytic activity, colony forming units (CFU), and total volatile basic nitrogen (TVB-N) were analyzed to study the quality changes in the raw materials at different days of storage (up to 3 days at 4 °C). After the silage treatment, the quality of the protein hydrolysates was evaluated by analysis of crude protein content, degree of hydrolysis (DH), amount of free amino acids (FAA), and TVB-N.

## 2. Material and Methods

### 2.1. Raw Material

Fresh saithe (*Pollachius virens*), with a total of 68 individuals (3468 ± 747 g in weight and 67 ± 5 cm in length), was provided to collect raw material for the experiment. The fish were caught by local fishers in the Trondheim fjord (63°30′ N/10°15′ E) in central Norway at a seawater temperature of 6 °C. The fish were caught during the spawning season in February 2021 due to the availability of fish migrating to the fjord in this period. The fish were immediately euthanized, bled out, and stored on ice until delivery to the laboratory facility. Further procedures were carried out by the researchers the same day.

Gutting of the fish was performed within 3 h after catch to collect fresh viscera (stomach, intestines, gonads, and liver) for the experiment. The raw material was divided into two groups: viscera containing all inner parts, including the liver (VL), and viscera not including the liver (V). The raw material in each group (7200 g) was homogenized with a HOBART (AE200) mincer (10 mm holes) and stored in the dark at 4 °C in a plastic bucket covered with plastic. Experimental data are shown in Table 1.

### 2.2. Experimental Overview

**Figure 1:**
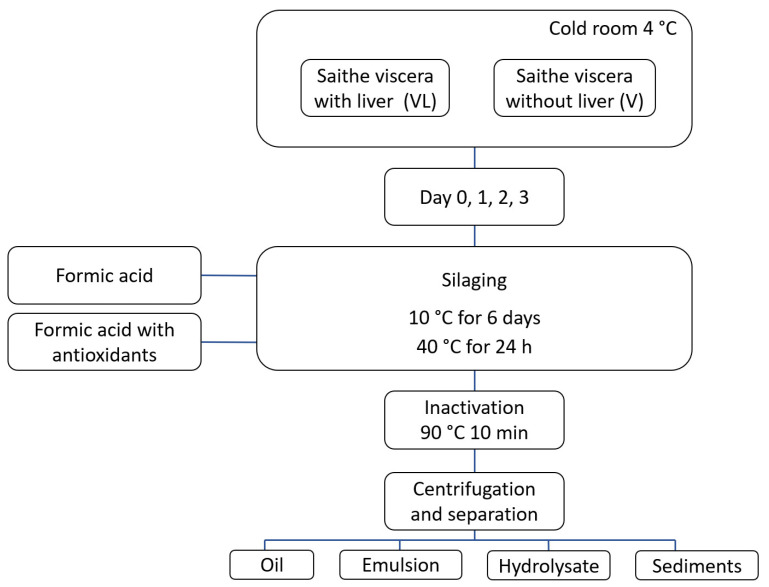
Experimental overview. Minced viscera with (VL) or without liver (V) were made from fresh raw material (day 0, 3 h after catch) or stored at 4 °C for 1, 2, or 3 days prior to silaging at 10 °C for 6 days, followed by 24 h at 40 °C to speed up the hydrolysis. VL and V were added with formic acid (VL-Silage, V-Silage) or formic acid containing antioxidants (VL-Silage-A, V-Silage-A). The silages were inactivated, centrifuged, and separated into fractions: oil, emulsion, hydrolysate, and sediments.

Minced raw material (1800 × 2) from each raw material group, i.e., viscera with (VL) and without (V) liver, were stored at 4 °C for 1, 2, and 3 days prior to the silaging treatment (Figure 1). On day 0, silages were made from fresh homogenized raw material (3 h after catch). The silages were made by reducing the pH in the mass to 3.8 with the addition of formic acid (28–34 mL) with antioxidants (SoftAcid Aqua MAB+; formic acid (85 % *w*/*w*), lignosulfonic acid (15 % *w*/*w*), antioxidant mixture; propyl gallate+ citric acid (0.35 % *w*/*w*), propylene glycol, butylated hydroxyanisole (BHA, 0.16 % *w*/*w*), Borregaard AS, Sarpsborg, Norway) or formic acid without antioxidants (SoftAcid Aqua M; formic acid (85 % *w*/*w*), lignosulfonic acid (15 % *w*/*w*)). Silaging treatments of VL and V with antioxidants were called VL-Silage-A and V-Silage-A, and silages without antioxidants were called VL-Silage and V-Silage. The silages were kept in 2 L glass beakers covered with plastic film in a dark room at 10 °C for 6 days. The silages were then transferred into 1 L Erlenmeyer flasks covered with aluminum foil and heated to 40 °C for 24 h on a heating cabinet under continuous stirring to speed up the hydrolysis. The pH was adjusted with formic acid each storage day to keep the pH at 3.8. After the silage treatment, each silage was heated to inactivate enzymes (90 °C for 10 min), centrifuged (2250× *g* for 10 min) in 1 L centrifuge bottles, and separated into the following fractions: oil, emulsion, protein hydrolysate, and sediments. Treatment times and temperatures were selected to simulate industrial fish silage preparation, ensuring adequate enzymatic activity to hydrolyze the raw material into a liquid silage product [26]. The protein hydrolysates were collected for further analyses. Prior to analysis, the protein hydrolysate was filtered through glass wool and freeze-dried for 3–5 days depending on the size of the sample. The silage treatments were performed in duplicate.

### 2.3. Dry Matter and Crude Protein

The content of dry matter in the hydrolysates was determined gravimetrically by heating the samples at 105 °C for 24 h [27]. The crude protein content was determined in dried hydrolysates via the Kjeldahl method with the use of an automated digester (KjeldDigester K-449, Büchi, Flawil, Switzerland) and a system for distillation and titration (KjelMaster K-375 and KjelSampler K-376, Büchi, Flawil, Switzerland). The protein content was calculated by multiplying the amount of nitrogen by a fish and meat factor of 6.25. The analyses were performed in duplicate.

### 2.4. General Proteolytic Activity and Water-Soluble Proteins

Raw material samples (10 g) were homogenized with distilled water (20 mL) using an Ultra Turrax (IKA, Staufen, Germany) for 20 s and centrifuged at 10,400× *g* for 20 min at 4 °C. The supernatants were filtered through glass wool, and the pH was measured. The general proteolytic activity was determined according to Barret [28] as described by Bergvik et al. [29]. The samples were diluted in phosphate–citric acid buffer with pH 3.8 or 7.0 and incubated at 40 °C for 60 min. The content of soluble peptides in the extracts and water-soluble proteins in untreated extracts were determined according to Lowry et al. [30]. The analyses were performed in triplicate. The proteolytic activity is presented as cut % wet weight based on the liberated (mg)/g wet weight of the trichloroacetic acid (TCA)-soluble peptides.

### 2.5. Bacterial Counts

Samples (5 g) were placed in 45 mL buffered peptone water (Bio-Rad, Hercules, CA, USA) and diluted in tryptone salt (Bio-Rad) until reaching a relevant concentration. Total viable aerobic bacterial counts were determined using Compact Dry TC (Nissui Pharmaceutical Co Ltd., Tokyo, Japan). The inoculated plates were incubated at 30 °C for 48 h. After incubation of the plates, the colonies were enumerated. Each sampling was performed in triplicate. Results are given as logarithms of colony-forming units per gram of the sample (Log CFU/g).

### 2.6. Total Volatile Basic Nitrogen

Total volatile basic nitrogen (TVB-N) was analyzed in raw material and hydrolysate samples according to Pearson [31] by using an Kjeltec 8100 distillation unit (FOSS Analytical, Shanghai, China). The samples (3 g of freeze-dried hydrolysate or 10 g of homogenized raw material) were transferred into 750 mL distillation tubes, mixed with 100 mL of water, and added to 1 g of magnesium oxide and 3 mL of paraffin oil as an antifoaming agent. Then, 25 g of boric acid (1%) ml was added to the receiver flasks, and samples were distillated with HCl. The results are presented as mg TVB-N/100 g, and the analysis was performed in duplicate.

### 2.7. Degree of Hydrolysis

Degree of hydrolysis (DH) was determined by a procedure proposed by Taylor [32] using an automatic titrator, as described by Kvangarsnes et al. [33]. The analyses were performed on freeze-dried hydrolysate, and 0.5 g of the sample was used instead of 1.5 g to ensure an optimal concentration for the reaction with the titrant. Results are presented as percentages of α-amino nitrogen out of the total nitrogen in the sample. The analysis was performed in duplicate.

### 2.8. Free Amino Acids

Determination of FAA content was carried out in freeze-dried hydrolysates according to the method of Osnes and Mohr [34]. A water-soluble extract was made of 0.25 g freeze-dried hydrolysate in 10 mL of double-distilled water. A sample of 1 mL of extract was transferred to a microcentrifuge tube with 0.25 mL 10% sulphosalisylic acid. Each sample was shaken and kept at 4 °C for 30 min, followed by centrifugation (10,000× *g*) for 10 min. The supernatant was diluted, filtered, and transferred (0.25 mL) into vials for analysis by HPLC. The analysis was conducted on a Dionex Ultimate 3000 HPLC (Thermo Fisher Scientific Inc., Waltham, MA, USA) coupled to a fluorescence detector. The amino acids were separated with the use of a NOVA-PAK C18 column (Waters Corporation, Milford, MA, USA). Methanol and 0.08 M sodium acetate with 2% tetrahydrofuran were applied and used as mobile phases with a flow rate of 0.9 mL/min. The analysis was performed in duplicate.

### 2.9. Statistical Analysis

SPSS software (version 29.0, IBM SPSS Statistics) was used for statistical analyses. One-way analysis of variance (ANOVA) and Tukey’s post hoc test were applied to determine significant differences between samples. Pearson’s correlation coefficient R was applied to evaluate the relationships between variables. The significance level was set to *p* < 0.05. The results are expressed as mean values of the samples ± standard deviations (SDs).

## 3. Results and Discussion

### 3.1. Properties and Quality of Raw Material

#### 3.1.1. pH, General Proteolytic Activity, and Water-Soluble Proteins

Both raw materials, minced viscera with (VL) and without (V) liver, had the same pH of 6.8 ± 0.1 on day 0 (fresh raw material). Similar pH values have been reported in previous studies on viscera from gadiform species [35,36,37]. However, out sorting of liver resulted in significantly higher pH values in V compared to VL on day 2 and 3 (Table 2). Storage led to increased pH values for both raw materials, with the highest pH measured on day 2 (VL: pH 7.0 ± 0.2, V: pH 7.2 ± 0.1), followed by a significant decrease on day 3 (VL: pH 6.5 ± 0.1, V: pH 6.8 ± 0.1). Liberation of content from the digestive tract and breakdown of stomach content (bones, krill etc.) is a possible explanation for the increase until day 2 and the higher pH found in V on the last days [37]. The decline in pH on day 3 might be explained by acid-producing bacteria present in the gut microbiota of fish [38].

The protein solubility, measured according to the amount of water-soluble (WS) proteins, relates to the proteolytic activity, as WS proteins, such as sarcoplasmic proteins, are liberated during the proteolytic breakdown of tissue cells [39]. The protein solubility increased significantly after 2 days of storage for VL and between each storage day for V, until day 2 (Table 2). On day 3, the WS protein in V declined and was significantly lower than on day 0–2. This could be explained by proteolytic breakdown of WS protein into smaller peptides and free amino acids [40].The amount of WS protein was significantly higher in V compared to VL, which was expected due to the higher protein content in V [41].

**Figure 2:**
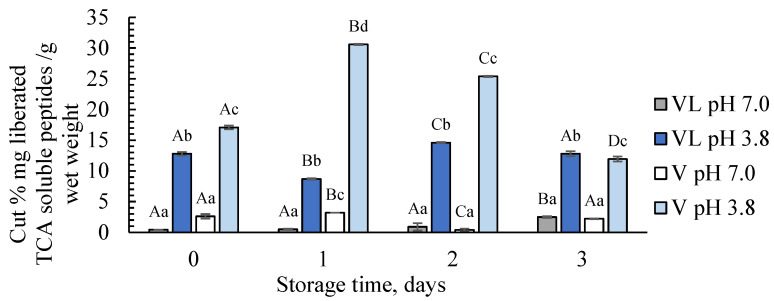
General proteolytic activity (cut % mg liberated TCA soluble peptides/g wet weight) in raw material of VL and V at different storage times (day 0–3) and pH (3.8 or 7.0). Significant differences (*p* < 0.05) are shown as different letters between days for the same sample (^A–D^) and between samples for the same day (^a–d^).

Determination of general proteolytic activity is a valuable tool to investigate a raw material’s susceptibility towards proteolytic hydrolysis under various conditions [40]. When comparing proteolytic activity in VL and V at the natural pH (pH 7), there was only a significant difference observed at day 1 of storage, with the highest activity in V (Figure 2). Under acidic conditions used for silaging (pH 3.8), the activity was significantly higher in V compared to VL on day 0–2. This is in accordance with previous studies on saithe, cod, and salmon [40,42,43], where higher proteolytic activity was found in viscera not containing liver compared to the liver. This indicates that more effective hydrolysis could be expected when the liver is sorted out prior to silaging. However, this was not observed at storage day 3, at which point VL had slightly higher activity compared to V. This might be explained by the fact that the reaction rates of proteases can change and decrease due to substrate availability, substrate exhaustion, and product inhibition [44]. As expected, reducing the pH to 3.8 highly increased the proteolytic activity (Figure 2). This can be explained by the high levels of pepsin in the stomach of fish, with an optimum pH of around 3.5 [45]. For VL, it can be expected that the lysosomal proteinases cathepsin D and E, located in the liver, also contribute, as these proteases have optimum pH values of around 3 [40].

#### 3.1.2. Quality of the Raw Material

Bacterial counts and total volatile basic nitrogen (TVB-N) were used as indicators to evaluate the quality of the raw material at different days of storage (day 0–3) prior to silaging. Bacterial counts, measured by colony-forming units (CFU), were slightly higher in viscera with liver (VL) compared to viscera without liver (V) (Table 3). However, this difference was only significant when comparing fresh raw materials (day 0). The storage time did not have a significant effect on bacterial counts, except the significantly higher counts in VL on day 0 compared to day 1–3. Although the raw material was minced and mixed to a homogenous mass, accumulation of particles of organs with high microbial activity, such as the intestines [46], in samples from VL on day 0 might explain the results. The bacterial counts were similar to the results (average of 4.6 ± 0.3 log CFU/g) reported by van’t Land et al. [22] in minced whole fish (frozen right after catch) used for silage production. Raeesi et al. [24] also reported similar counts of 5.3 log CFU/g in fresh minced trout viscera. Overall, the bacterial counts indicate that the raw materials were of acceptable microbial quality throughout the storage period, as the results were below the quality guidelines for seafood products (<7 log CFU/g) [47].

Total volatile basic nitrogen (TVB-N), which is mainly the sum of trimethylamine (TMA) and ammonia (NH_3_), is an important indicator of the freshness of raw material used for fish silage production [20,48] and relates to the microbial and enzymatic degradation of proteins and amines [49]. TVB-N values in the raw materials were significantly higher in V compared to VL on day 0 and 1 (Table 3). However, no significant difference was observed on day 2, and on day 3, a significantly higher value was found in VL. For both raw materials, the TVB-N levels dropped from day 0 to day 1, where the values were significantly lower than the other days. This might be explained by the reduction in amide nitrogen that were observed on the first days of storage of fish in previous studies [50,51]. The highest values were observed in raw materials stored for 3 days. Haaland and Njaa [51] reported that TVB-N values accelerated from day 3 to day 7 during storage of capelin. This was explained by the degradation of α-amino groups in amino acids to NH_3_. The significant increase in TVB-N in VL observed from day 2 to day 3, where the bacterial counts stayed stabile, could indicate NH_3_ formation due to proteolytic activity rather than microbial release of TMA.

The TVB-N values exceeded the limit (35 mg TVB-N/100 g) set for fishery products intended for human consumption (*Gadidae* species), but were close to maximum levels (60 mg TVB-N/100 g) for whole fishery products used directly for the preparation of fish oil for human consumption at days 0–2 [52]. However, the limit for fish oil production is based on evaluations of the freshness of whole fish, and generally higher values would be expected for viscera. Overall, the TVB-N levels were below the recommendations for raw material intended for fishmeal production (80 mg/100 g) [53], except the raw material of VL on day 3.

### 3.2. Characteristics of Protein Hydrolysates

#### 3.2.1. Yield and Protein Content

Four fractions were obtained from the lipid-rich silages (VL-S, VL-S-A) after heat inactivation and centrifugation: oil, emulsion, hydrolysate (water-soluble protein phase), and sediment. Two fractions of hydrolysate and sediment were obtained from silages made from raw material without liver (V-S, V-S-A). Results regarding the proximate composition of raw material, as well as the composition and quality of lipids in raw material, oil, and sediment fractions obtained from silages with liver in the present experiment, have previously been published by Meidell et al. [41].

**Figure 3:**
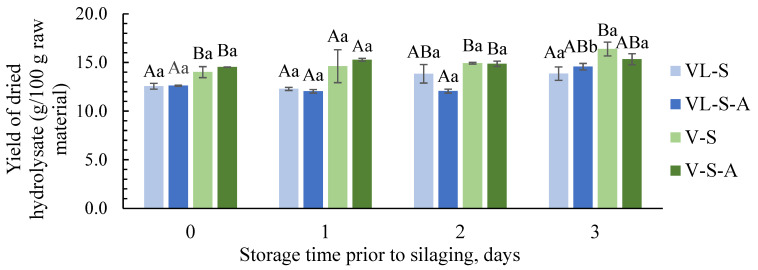
Yield of dried hydrolysate (g/100 g raw material) obtained from different silages made from raw materials with different freshnesses (storage for 0–3 days prior to silaging). Different letters (^A,B^) indicate significant (*p* < 0.05) differences between silage groups (VL-S, VL-S-A, V-S, V-S-A) with the same freshness (storage day 0, 1, 2 or 3) or significant (*p* < 0.05) differences (^a,b^) between different freshnesses (storage day 0, 1, 2 and 3) for the same silage groups (VL or V).

During hydrolysis, it is generally desired to optimize the hydrolysate yield while minimizing emulsion and sediment fractions [54]. The highest hydrolysate yields were obtained when livers were sorted out prior the silage treatment (V-S, V-S-A, Figure 3). This was expected, as these silages were made from raw material with a higher protein content [41], which was in accordance with results reported by Slizyte et al. [37] during enzymatic hydrolysis of cod viscera and backbones. In general, slightly higher yields seemed to be obtained when the raw materials were stored prior to silaging compared to fresh. This can be explained by increased liberation of highly soluble peptides and free amino acids during proteolysis [15]. However, a slight decrease in yield on days 1 and 2 was observed for VL-S-A. Changes in peptide sizes might explain these results, as this influences their interfacial/surface properties and their ability to form emulsions [55]. This likely affected the distribution of peptides in the different fractions. The decreases on days 1 and 2 could be due to a larger proportion of large-sized peptides, which are effective emulsifiers and, thus, end up in the sludge or emulsion fractions. The increase on day 3 can be attributed to more extensive proteolysis resulting in a higher proportion of soluble, small-sized peptides and free amino acids with limited capacity to form emulsions. There was no clear trend reflected by the addition of antioxidants.

High-quality hydrolysates are characterized by a high protein content [15], which is directly related to pricing in the industry [56]. According to Chalamaiah et al. [57], protein-rich hydrolysates contain 60–90% protein. In the present study, dried hydrolysates had generally high contents of crude protein, ranging between 75 and 81% (Table 4). Slizyte et al. [54] and Jafarpour et al. [58] reported similar results in dried hydrolysates obtained after enzymatic hydrolysis of cod viscera (68–84%) and cod frames and meat (76–84%). The results were also similar to the protein content found in commercial cod protein hydrolysate intended for human consumption (75%) [59]. Additionally, the hydrolysates were rich in protein compared to what was found (62%) in fish meal produced from viscera and heads on board a Norwegian whitefish trawler [60].

The lipid content in the raw material used for hydrolysis is highly relevant, as a higher content tends to reduce the protein content in the hydrolysate due to the formation of lipid–protein complexes [61,62]. In our previously published paper [41], we analyzed the lipid content in raw materials and found that viscera with liver (VL) contained, on average, 13% more lipids than the viscera without liver (V) [41]. Thus, it could be expected that hydrolysates made from VL would have lower protein contents compared to those made from V. However, the sorting of liver did not seem to significantly affect the protein contents of the hydrolysates (Table 4). This indicates that the lipids originating from the liver were efficiently separated into the oil and sediment fractions.

Raw material freshness affected the protein content in the hydrolysates significantly, with the highest contents (80–81%) obtained after silaging of fresh raw material (0 days of storage). For hydrolysates obtained from three of four silage groups, a slight protein decrease was observed between each storage day. For all groups, the most significant decline in protein content was observed when the raw material was stored for 3 days (75–77% protein). This resulted in a loss of 4–5% protein in the hydrolysates obtained from silages with liver (VL-S, VL-S-A), and a 3–4% decline in viscera hydrolysates (V-S, V-S-A) compared to silages of fresh raw material. Slizyte et al. [54] found that phospholipids constituted the major lipid class in hydrolysates obtained from cod viscera. It is likely that enzymatic liberation of phospholipids and peptides with good emulsifying properties resulted in the formation of protein–lipid complexes ending up in the hydrolysates after longer raw material storage [54,55]. This could also be supported by the slightly higher protein loss observed for the most lipid-rich silages (VL-S, VL-S-A). An increased tendency for lipids and proteins to form complexes after longer storage was also supported by previous findings showing that an emulsion layer tends to appear when viscera is stored for 3–4 days at 4 °C prior to processing [9,41].

Decreases in the protein content were also observed in the silage product during storage [22,24,63,64]. This was explained by hydrolysis and conversion of proteins into volatile nitrogen and ammonia (NH_3_) [22,24]. According to Nørgaard et al. [65], the protein content in silage is likely to change during storage due to hydrolysis of protein into smaller protein fragments, peptides, and free amino acids. The results in the present study’s result showing that changes in protein content can also be expected in the resulting hydrolysates and that this is not only related to the silaging process, but also to the raw material used.

The presence of antioxidants is also likely to affect proteins during storage and processing, as lipid oxidation can cause lipid–protein interactions; promote protein oxidation; change the structures of protein, peptides, and amino acids; and influence the accessibility of peptide bonds for proteases [16]. However, in this current study, silaging with antioxidants did not seem to affect the protein content in the hydrolysates significantly.

#### 3.2.2. Degree of Hydrolysis

The degree of hydrolysis (DH) reflects the amount of hydrolyzed peptides and is closely related to the properties of hydrolysates, including solubility and molecular weight distribution [39]. DH was analyzed in dried hydrolysates obtained after silaging and ranged between 44 and 57% (Table 4). Silaging of fresh raw materials led to the lowest DH values (44–46%). Opheim et al. [62] found a similar DH of 48% in dried hydrolysates obtained from salmon viscera after hydrolysis by endogenous enzymes. Sajib et al. [23] also reported a similar DH of 43% in herring silage (containing viscera) after 7 days of silaging at 12 °C. However, these studies used the o-phthaldialdehyde assay (OPA) method, while formol titration was used in the present study. Direct comparison of the results obtained using different DH methods is difficult, as the results tend to be highly dependent on the method used [66]. According to Espe and Lied [63], DH is highly dependent on the raw material composition, and the highest DH was found in silages made from enzyme-rich raw material such as viscera. However, no significant effect of sorting was found when comparing silages made at storage day 0 and 1 in the present study. For silages made on day 3, silages with liver (VL-S and VL-S-A) had significantly higher DH compared to those without liver (V-S and V-S-A). These results did not reflect the proteolytic activity measured in the raw materials, where viscera without liver (V) had generally higher activity (day 0–2, Figure 2). As previously mentioned, the reaction rates of proteases might change due to substrate availability, substrate exhaustion, and product inhibition [44]. Such changes, in addition to differences in substrate specificity [39] and the content and composition of proteins in the raw materials, likely influenced the hydrolysis during silaging and the resulting DH. In addition, studies have reported a generally high proteolytic activity in the livers of saithe and cod at acidic pH levels [40,43].

In hydrolysates from silages with liver (VL-S, VL-S-A), the DH increased significantly when the raw material was stored for 2 days or longer (Table 4). For all silage groups, storage of raw material for 3 days led to hydrolysates with significantly higher DH (52–57%) compared to the other days. The most important increase was between day 2 and 3, where the DH values increased by 7–9%. Significant increases in DH have also been observed in silage products after longer periods of storage in studies on saithe and cod [63], whiting [22], and herring [14,23]. This can be explained by the increased availability of substrates for proteases when the viscosity decreases, as well as more time for the proteases to be active.

It is well-known that lipid oxidation might influence the accessibility of peptide bonds for proteolytic enzymes [16]. In the present study, the DH in the hydrolysates did not seem to be significantly affected by addition of antioxidants during silaging. Still, a slightly lower DH was obtained when antioxidants were added to the most lipid-rich silages (VL-S, VL-S-A). Since it is known that reactions between lipid oxidation products and amino acids can act as inhibitors of proteolytic enzymes [67], an opposite trend could be expected. However, the results were in accordance with a study by Sajib et al. [25] in which herring silages with antioxidants had lower DH compared to the controls.

#### 3.2.3. Free Amino Acids

The amount of total free amino acids (FAAs) is closely related to DH, as FAA is liberated during proteolytic hydrolysis of peptide bonds. There was a significant, strong, positive relationship between DH and amount of FAA for hydrolysates obtained from VL-S (r = 0.970), VL-S-A (r = 0.900), and V-S (r = 0.903), and a significant moderate positive correlation for V-S-A (r = 0.664). Correlations between DH and FAA have also been demonstrated in previous studies [23,61,62,68]. It is generally desired to limit the amount of FAAs, as they are less absorbed than peptides and intact proteins [15]. The content of total FAAs in the hydrolysates increased from 137–145 mg/g dry sample in silages made from fresh raw material to 153–179 mg/g in silages made on day 3 (Table 4). Similar results were reported by Slizyte et al. [54], who found 181 mg FAA/g dried hydrolysate after endogenous hydrolysis of cod viscera.

**Figure 4:**
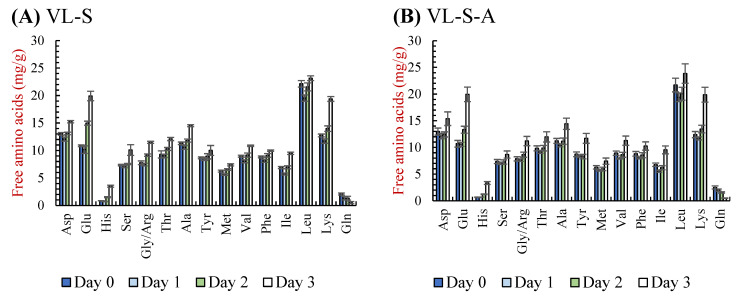

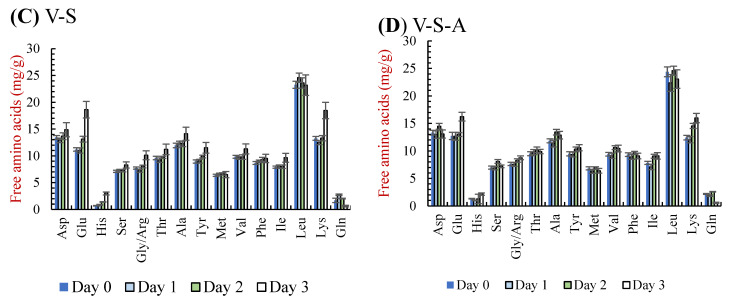
Composition of free amino acids (mg/g): aspartic acid (asp), glutamic acid (Glu), histidine (His), serine (Ser), glycine/arginine (Gly/Arg), threonine (Thr), alanine (Ala), tyrosine (Tyr), methionine (Met), valine (Val), phenylalanine (Phe), isoleucine (Ile), leucine (Lau), lysine (Lys), and glutamine (Gln) in dried hydrolysates obtained from different silage groups (viscera with liver silage: VL-S, viscera with liver silage with antioxidants: VL-S-A, viscera silage: V-S, viscera silage with antioxidants: V-S-A) related to raw material freshness (storage for 0–3 days). Asparagine (Asn) is not shown due to the low content (<0.05 mg/g) found in the samples. Results are shown for hydrolysates obtained from different silage groups: (**A**) Viscera with Liver Silage, (**B**) Viscera with Liver with Antioxidants, (**C**) Viscera Silage and (**D**) Viscera Silage with Antioxidants.

Analysis of the composition of FAA revealed that leucine (Leu), aspartic acid (Asp), lysine (Lys), glutamic acid (Glu), and alanine (Ala) were the dominating FAAs in the hydrolysates (Figure 4). Opheim et al. [62] reported a similar FAA composition in hydrolysates obtained after endogenous hydrolysis of salmon viscera. The results were also in accordance with the most abundant amino acids found in silage or silage hydrolysates obtained from saithe viscera [69], cod viscera [63], herring residuals containing viscera [14], and fishmeal produced from whitefish viscera [70].

Compared to silaging of fresh raw materials, 3 days of storage prior to the treatment led to a significant increase in free glutamic acid and lysine in VL-S, VL-S-A, and V-S hydrolysates (Figure 4). Glutamic acid is one of the main amino acids found in the viscera of codfish [63,71], and is thus available and likely to be released during proteolysis after longer storage. This was also in accordance with a study by Farvin et al. [59], which found Glu to be the predominant FAA in commercial cod hydrolysate. Increases in total lysine have also been observed in the hydrolysate phase after storage of silage made from saithe viscera [69]. The increase in free lysine in the present study indicates tryptic hydrolysis, as trypsin is specific towards lysine [39]. Trypsin is responsible for the main proteolytic activity in the viscera of codfish at neutral pH levels [72], and is inactive during silaging, where pepsins, and to some degree cathepsins, are responsible for the hydrolysis [15]. This indicates that the raw material’s freshness and the proteolytic activity in the raw material prior to silaging can be reflected in the FAA composition in the resulting hydrolysates.

For all silage groups, a significant decrease in free glutamine (Gln) in the hydrolysates was observed when silage was made on day 3 compared to day 0. Opheim et al. [62] observed a 97% decrease in free glutamine in hydrolysates produced with formic acid (pH < 4) compared to lab-scale hydrolysates without the addition of acid. Haaland and Njaa [50] also reported a decrease in free glutamine in fish silage and demonstrated that ammonia formation from degradation of the amide group of glutamines caused the decrease.

#### 3.2.4. Total Volatile Basic Nitrogen

There was a significant strong correlation between TVB-N in raw materials and that in the corresponding dried hydrolysates obtained from all silage groups: VL-S (0.905), VL-S-A (0.964), V-S (0.902), and V-S-A (0.726). This was in accordance with a study by Haaland and Njaa [51] which reported that TVB-N in silages of capelin reflected the levels found in the raw material. For silages made from fresh raw material (day 0), the values in the hydrolysates ranged between 131 and 142 mg TVB-N/100 (Table 4). This was similar to what has been reported (137 mg TVB-N/100 g) in fish meal of mackerel and herring cut-offs processed 1–3 days after catch [56]. Nguyen et al. [73] found similar TVB-N levels (140 TVB-N/100 g) in fishmeal produced from viscera containing raw fish material with a similar TVB-N value as that in the present study (68 mg TVB-N/100 g). The results did not show any clear trend reflected by sorting of the raw material. For hydrolysates obtained from silages of raw material with liver (VL-S, VL-S-A), significantly higher TVB-N values were obtained when the raw material was stored from 2 days or more. For all groups, significantly higher values were observed when silaging started on day 3 compared to days 0–2; they leveled up to 151–179 mg TVB-N/100 g. This was in accordance with a study by Opstvedt et al. [74], which found increased levels of TVB-N in herring fish meal when raw material was stored for 9 days compared to 1 day at 10–15 °C.

As mentioned previously, TVB-N mainly consists of TMA and NH_3_. Previous studies [26,48] have indicated that during silaging, TMA, which is produced during the microbial degradation of trimethylamine oxide (TMAO), stays stable, and an increase in TVB-N is caused by NH_3_ formation during autolysis. This was also demonstrated by van’t Land et al. [22], who reported an increase in TVB-N while TMA-N values remained stable during silage storage. According to Haaland and Njaa [50] and Espe et al. [19], formation of NH_3_ from the amide groups of glutamine, and, to some degree, asparagine, during autolysis explains the increases in TVB-N during silaging [19]. Although the total glutamine content was not analyzed in the present study, significant decreases in free glutamine were observed in the hydrolysates made on the last storage day (day 3) (Section 3.2.3, Figure 4). This might indicate the formation of NH_3_ from this amino acid.

Studies have also related increases in TVB-N to decreases in crude protein content in silage [22,24], which is explained by the release of NH_3_ during hydrolysis. A significant, strong, and negative correlation was found between the amount of TVB-N and the protein content in the hydrolysates in the present study: VL-S (−0.941), VL-S-A (−0.892), V-S (−0.794), and V-S-A (−0.864). However, this might also be attributed to the distribution of protein and other nitrogenous compounds into other fractions during separation. Overall, the addition of antioxidants did not seem to affect the TVB-N values, which was in accordance with a study by Sajib et al. [25] which found no significant effect of antioxidant addition on TVB-N in herring silage.

## 4. Conclusions

This study demonstrated that protein-rich hydrolysates could be obtained after silaging of saithe viscera. While out sorting of the liver resulted in slightly higher hydrolysate yields, it did not significantly impact the protein content or the overall quality of the hydrolysates. Preservation of the raw material shortly after catch resulted in optimized quality and higher protein content in the hydrolysates compared to longer storage of the raw material. Storage for 2–3 days prior to silaging resulted in significant increases in DH, FAA, and TVB-N levels in the resulting hydrolysates. The most notable changes were observed between days 2 and 3 of storage, while 1 day of storage did not reduce the quality of the hydrolysates. This suggests that the raw material does not necessarily need to be processed immediately after catch, which improves flexibility for the fishing vessel. In addition, the results demonstrated a limited effect on the studied quality parameters by using antioxidants during silaging, indicating that lipid oxidation might not strongly affect the proteins or proteolytic activity during the treatment.

This approach does not only enhance the efficiency of the silage process, but could also promote increased sustainability by maximizing the use of available raw material, particularly if the liver quality is insufficient for separate oil production. However, it must be noted that the present work was conducted on a laboratory scale and may not fully replicate industrial-scale conditions. Variations in the composition of viscera due to factors like the season, maturity stage, and fishing ground could also influence the results. Additionally, the findings for saithe viscera might not apply universally to other whitefish species. Further research should focus on validating the results on an industrial scale, exploring the effects of biological variations, and comparing outcomes across different species.

## Figures and Tables

**Table 1:** Raw material data (mean ± SD) of raw material used in the experiment: viscera with liver (VL) and viscera without liver (V).

Experimental Data	Viscera with Liver (VL)	Viscera without Liver (V)
Number of fish	27	41
Fish length, cm	66 ± 5	67 ± 5
Round weight, g	3388 ± 809	3521 ± 709
Viscera with liver, g	593 ± 257	612 ± 209
Viscera without liver, g	445 ± 245	485 ± 173
Liver, g	123 ± 45	127 ± 56

**Table 2:** pH and amount of water-soluble (WS) protein (% of wet weight) in the raw material of VL and V at different storage times (day 0–3).

Viscera with Liver (VL)				
Parameter	Day 0	Day 1	Day 2	Day 3
pH	6.8 ± 0.1 ^Aa^	6.9 ± 0.0 ^Aab^	7.0 ± 0.2 ^Ab^	6.5 ± 0.1 ^Ac^
WS protein, %	4.9 ± 0.3 ^Aa^	5.1 ± 0.0 ^Aa^	6.9 ± 0.2 ^Ab^	6.9 ± 0.0 ^Ab^
**Viscera (V)**				
Parameter	Day 0	Day 1	Day 2	Day 3
pH	6.8 ± 0.1 ^Aa^	6.8 ± 0.1 ^Ba^	7.2 ± 0.1 ^Bb^	6.8 ± 0.1 ^Ba^
WS protein, %	9.5 ± 0.2 ^Ba^	10.6 ± 0.1 ^Bb^	12.3 ± 0.3 ^Bc^	8.5 ± 0.3 ^Bd^

Different letters (^A,B^) indicate significant (*p* < 0.05) differences between raw materials (VL and V) with the same freshness (storage day 0, 1, 2, or 3) or significant (*p* < 0.05) differences (^a–d^) between different freshnesses (storage day 0, 1, 2, and 3) of the same raw material (VL or V).

**Table 3:** Bacterial count (Log CFU/g) and TVB-N values (mg/100 g) in viscera with and without liver (VL, V) on different days of storage.

Viscera with Liver (VL)				
Parameter	Day 0	Day 1	Day 2	Day 3
Bacterial count (Log CFU/g)	5.7 ± 0.1 ^Aa^	2.4 ± 0.1 ^Ab^	3.2 ± 0.3 ^Ab^	3.3 ± 0.8 ^Ab^
TVB-N (mg/100 g)	60.6 ± 1.5 ^Aa^	49.2 ± 2.2 ^Ab^	62.1 ± 1.7 ^Aa^	83.2 ± 1.2 ^Ac^
**Viscera (V)**				
Parameter	Day 0	Day 1	Day 2	Day 3
Bacterial count (Log CFU/g)	2.6 ± 0.1 ^Ba^	2.4 ± 0.3 ^Aa^	2.6 ± 0.3 ^Aa^	3.2 ± 0.5 ^Aa^
TVB-N (mg/100 g)	66.2 ± 2.1 ^Bac^	54.7 ± 1.2 ^Bb^	61.5 ± 2.2 ^Aa^	71.7 ± 0.2 ^Bc^

Different letters (^A,B^) indicate significant (*p* < 0.05) differences between raw materials (VL and V) with the same freshness (storage day 0, 1, 2 or 3). Different letters (^a–c^) indicate significant (*p* < 0.05) differences between different freshnesses (storage day 0, 1, 2 and 3) for the same raw material (VL or V).

**Table 4:** Crude protein (%, mean ± SD), degree of hydrolysis (%, mean ± SD), total free amino acids (mg/g dry sample, mean ± SD), and total volatile basic nitrogen (mg/100 g dry sample, mean ± SD) in dried hydrolysates obtained through silaging of raw materials with different freshnesses. Days 0–3 indicate days of raw material storage prior to silaging.

VL-S (Viscera with Liver Silage)				
Parameter	Day 0	Day 1	Day 2	Day 3
Crude protein, %	79.8 ± 0.5 ^Aa^	79.7 ± 0.3 ^Aa^	79.9 ± 0.5 ^Aa^	74.7 ± 0.6 ^Ab^
TVB-N, mg/100 g	131.1 ± 0.5 ^Aa^	134.3 ± 1.2 ^Aa^	152.2 ± 0.8 ^Ab^	178.6 ± 0.4 ^Ac^
DH, %	46.4 ± 0.7 ^Aa^	45.5 ± 0.3 ^Aa^	47.8 ± 0.5 ^Ab^	57.0 ± 0.2 ^Ac^
FAA, mg/g	137.5 ± 2.9 ^Aa^	125.6 ± 4.2 ^Ab^	146.0 ± 4.0 ^ABc^	177.7 ± 2.4 ^Ad^
**VL-S-A (Viscera with Liver Silage with Antioxidants)**				
Parameter	Day 0	Day 1	Day 2	Day 3
Crude protein, %	80.1 ± 0.8 ^Aa^	79.1 ± 0.3 ^Bab^	77.8 ± 1.0 ^Bb^	75.8 ± 0.2 ^ABc^
TVB-N, mg/100 g	137.0 ± 0.6 ^Ba^	132.6 ± 0.7 ^Ab^	146.0 ± 0.9 ^Bc^	179.7 ± 0.7 ^Ad^
DH, %	45.5 ± 0.6 ^Aa^	44.7 ± 0.7 ^Aa^	47.6 ± 0.7 ^Ab^	55.5 ± 0.4 ^Bc^
FAA, mg/g	136.7 ± 6.0 ^Aa^	126.4 ± 2.2 ^Aa^	136.7 ± 7.2 ^Aa^	179.4 ± 13.4 ^Ab^
**V-S (Viscera Silage)**				
Parameter	Day 0	Day 1	Day 2	Day 3
Crude protein, %	80.6 ± 0.2 ^Aa^	79.3 ± 0.3 ^ABb^	79.0 ± 0.1 ^ABb^	76.8 ± 0.8 ^BCc^
TVB-N, mg/100 g	142.3 ± 0.9 ^Ca^	126.1 ± 0.6 ^Bb^	134.4 ± 0.4 ^Cc^	177.1 ± 1.4 ^ABd^
DH, %	45.2 ± 0.4 ^Aa^	44.8 ± 0.3 ^Aa^	46.1 ± 0.1 ^Ba^	52.7 ± 0.9 ^Cc^
FAA, mg/g	141.7 ± 4.0 ^Aa^	142.9 ± 3.7 ^Ba^	148.0 ± 5.6 ^ABa^	171.0 ± 14.4 ^ABb^
**V-S-A (Viscera Silage with Antioxidants)**				
Parameter	Day 0	Day 1	Day 2	Day 3
Crude protein, %	80.3 ± 0.3 ^Aa^	79.0 ± 0.2 ^Bb^	78.6 ± 0.2 ^ABb^	77.3 ± 0.5 ^Cc^
TVB-N, mg/100 g	136.9 ± 0.4 ^Ba^	138.7 ± 1.2 ^Ca^	139.0 ± 2.2 ^Ca^	174.9 ± 0.5 ^Bb^
DH, %	44.4 ± 0.3 ^Aa^	44.7 ± 0.4 ^Aa^	48.6 ± 0.3 ^Ab^	52.4 ± 0.6 ^Cc^
FAA, mg/g	145.3 ± 5.4 ^Aab^	139.6 ± 7.5 ^Ba^	157.1 ± 4.8 ^Bb^	152.9 ± 6.6 ^Bab^

Different letters (^A–C^) indicate significant (*p* < 0.05) differences between different silage groups (VL-S, VL-S-A, V-S, and V-S-A) made from raw material with the same freshness (storage for 0, 1, 2 or 3 days prior to silaging). Different letters (^a–d^) indicate significant (*p* < 0.05) differences between silages made from raw material with different freshnesses (storage for 0, 1, 2 and 3 days prior to silaging) within the same silage group (VL-S, VL-S-A, V-S or V-S-A).

## Data Availability

The original contributions presented in the study are included in the article, further inquiries can be directed to the corresponding author.
